# Dietary effects of protected fat, soybean meal, and heat-treated soybean meal on performance, physiological parameters, and behavioral measurements of early-fattening Hanwoo steers under heat stress conditions

**DOI:** 10.5713/ab.24.0236

**Published:** 2024-10-24

**Authors:** Jun Sik Woo, Sun Sik Jang, Jeong Hoon Kim, Hong Gu Lee, Keun Kyu Park

**Affiliations:** 1 Department of Animal Science and Technology, Konkuk University, Seoul 05029, Korea; 2 National Institute of Animal Science, Rural Development Administration, Pyeongchang 25340, Korea; 3 Cargill Agri Purina Inc., Seongnam 13630, Korea

**Keywords:** Fattening Period, Hanwoo Steers, Heat Stress, Heat-treated Soybean Meal, Protected Fat, Rumen Undegradable Protein

## Abstract

**Objective:**

This study evaluated the effects of increased levels of dietary total digestible nutrient (TDN) and crude protein (CP) using protected fat (PF), soybean meal (SB), and heat-treated soybean meal (HSB) on performance, physiological parameters, and behavioral measurements of early-fattening Hanwoo steers under heat stress conditions.

**Methods:**

Thirty-six steers (480.9±58.6 kg, 15.9±1.4 months) were assigned to 4 treatments: control (TDN 75%, CP 15%, rumen degradable protein [RDP]:rumen undegradable protein [RUP] = 62:48); PF (TDN 82.5%, CP 15%, RDP:RUP = 62:48); PF+SB (TDN 82.5%, CP 16.5%, RDP:RUP = 62:48); and PF+SB+HSB (TDN 82.5%, CP 16.5%, RDP:RUP = 48:52) for a total of 16 weeks with division of 4 weeks. The average temperature-humidity index (THI) was 82.9 (1st; moderate), 76.9 (2nd; mild), 70.9 (3rd; comfort), and 65.8 (4th period; comfort).

**Results:**

Dry matter intake during whole period did not differ among treatments but decreased by 34% and 19%, respectively during 1st and 2nd compared to 4th. Average daily gain (ADG) of PF+SB+HSB was higher (p<0.05) than that of control during the 1st period, and those of both PF+SB and PF+SB+HSB were higher (p<0.05) than Control during the 2nd. The ADG during whole period was in the order of PF+SB+HSB (1.23), PF+SB (1.18), PF (1.11), control (0.98 kg/d) (p<0.05). As THI increased, rectal temperature and all blood parameters increased, while blood glucose levels decreased (p<0.05). Behavioral changes during 1st period compared to 3rd included decreases in lying (24%), walking (48%), and eating (40%), and increases in total standing (50%) and drinking (43%) (p<0.05). Rumination during standing was 38% higher, and rumination during lying was 32% lower (p<0.05).

**Conclusion:**

This study demonstrates 10% of increased levels of dietary TDN using PF and CP considering RUP can prevent performance reduction in early-fattening of heat-stressed Hanwoo steers and have positive effects on performance recovery from post-heat stress.

## INTRODUCTION

Heat stress in ruminants negatively affects performance, such as dry matter intake (DMI), average daily gain (ADG), and feed conversion ratio (FCR) [1]. In order to efficiently increase heat dissipation, it is necessary to increase blood volume in peripheral tissue and respiratory rate [2]. The energy requirement for maintenance increases by 7% during shallow breathing and by 11% to 25% during heavy panting in ruminants [3]. Thus, the National Institute Animal Science of Korea (NIAS) [4] recommends increasing the energy requirement for maintenance to 110% under heat stress conditions that exceed the upper critical temperature threshold, i.e. above moderate-severe boundary (THI 84 to 85).

Studies on the decrease in performance and changes in physiological parameters of Hanwoo due to heat stress are very limited [5–7]. Ruminants under heat stress consume energy through various metabolic processes to maintain homeostasis, which negatively affects performance [4]. Moreover, heat stress reduces rumen motility and activity, slowing the passage of digesta through the gastrointestinal tract, which results in a decrease in feed intake, acetate to propionate ratio and pH in the rumen [8]. Therefore, additional feeding of rumen-protected energy and protein should mitigate these negative effects under heat stress.

In previous research [9], late-fattening Hanwoo steers under heat stress conditions were fed diets with a 10% increase in total digestible nutrient (TDN) and crude protein (CP) contents compared to Control. Rumen-protected fat was used to increase the TDN level, but the CP source was a general soybean meal (SB) with low rumen undegradable protein (RUP) content. The results showed that the treatment with increased levels of both TDN and CP by 10% did not show any significant differences in performance or carcass grades compared to the treatment that only increased the TDN level by 10%, and the reason is postulated to solely increasing CP levels without considering RUP. Therefore, in this study, in situ ruminal degradability and *in vitro* intestinal digestibility of all feed ingredients used in the total mixed ration (TMR) diet were measured prior to *in vivo* animal trial. Based on this, the study aimed to assess the effects of increasing levels of both TDN and CP considering RUP by 10% using rumen-protected fat (PF) and heat-treated soybean meal (HSB) on performance, physiological parameters, and behaviors in Hanwoo steer during early-fattening period under heat stress conditions.

## MATERIALS AND METHODS

### Animal care

The experimental protocol was reviewed and approved by the Institutional Animal Care and Use Committee at Konkuk University (Approval number: KU22052 [*in situ*] and KU22053 [*in vivo*]).

### Chemical analysis

All samples of feed ingredients and experimental diets for chemical analysis were dried in a forced-air oven connected to the sucking fan for over 24 h at 60°C and ground using a Wiley mill (Model 4; Thomas Scientific, Swedesboro, NJ, USA) through a 2 mm screen. These samples were analyzed to dry matter (DM; method 930.15), CP (method 976.05), ether extract (EE; method 2003.05), ash (method 942.05), crude fiber (method 962.09), neutral detergent-insoluble fiber (NDF; method 2002.04), acid detergent-insoluble fiber (ADF), and lignin (method 973.18) as described in AOAC [10].

The concentrations of amino acids in the feed ingredient samples were determined using ion-exchange chromatography followed by post-column derivatization with ninhydrin. Prior to the analysis, the samples underwent cold performic acid oxidation overnight to convert methionine and cysteine into methionine sulfone and cysteic acid, respectively, in preparation for hydrolysis.

### *In situ* ruminal dry matter and crude protein degradability

All feed ingredients for the experimental TMR diets used in the *in vivo* trial were measured for *in situ* ruminal DM and CP degradability and *in vitro* pepsin-pancreatin CP digestibility to derive precise RUP values. Two Holstein dairy cows (465±17.2 kg) fitted with a ruminal cannula were fed 4 kg of rice straw hay and 3 kg of formula feed daily at 09:00 and 18:00. The animals had free access to fresh water and mineral blocks. Rumen degradability of the feed ingredients was assessed by filling approximately 4 g of sample into nylon bags (10×20 cm [width×height], pore size 50 μm; R1020; Ankom Technology, Macedon, NY, USA). The ratio of sample size to bag surface area was maintained at 10 mg/cm^2^, based on the range (10 to 20 mg/cm^2^) suggested by Nocek [11]. Feed samples were incubated in triplicate for each ruminal incubation time.

The incubation times were set at 0, 4, 8, 16, 24, 48, and 72 h, with bags containing feed samples introduced into the ruminal ventral sac immediately after the morning feeding. The schedule of incubation time employed the ‘gradual addition/gradual out’ method [12] to minimize the number of nylon bags incubated in the rumen, thus limiting the maximum number of bags at each incubation time to less than 45. After incubation, the nylon bags were immediately rinsed in cold tap water until the water ran clear, and then dried in a 65°C dry oven for 48 h. Dried nylon bags were cooled in a desiccator before their weights were measured. Subsequently, the nylon bag residues of the same sample from each incubation time were collected and analyzed for CP according to AOAC [10].

The degradability of DM from the nylon bag method was modeled using an exponential function, and the effective degradability (ED) were calculated based on the equations proposed by Ørskov and McDonald [13]:


$$\begin{array}{l} P_{1}=a+b(1-e^{-ct})\\ \text{ED}=a+b\times c/(c+k) \end{array}$$

where, *P*_1_ represents the DM degradability at time *t*, *a* is the fraction that degrades rapidly, *b* is the fraction that is insoluble but potentially degradable, *c* is the rate for the *b* fraction, *t* is the time the feed remains in the rumen suspension, and *k* represents the outflow rates from the rumen, with an assumed outflow rate (*k*) of 0.02 per hour.

Subsequently, the percentages of rumen degradable protein (RDP) and RUP (% of CP) of feed ingredients can be calculated as follows, based on the guidelines from National Research Council (NRC) [3]:


$$\begin{array}{l} P_{2}=A+B(1-e^{-kd\times t})\\ C=100-(A+B)\\ \text{RDP}=A+B[kd/(kd+kp)]\times 100\\ \text{RUP}=B[kp/(kd+kp)]+C\times 100 \end{array}$$

where, *P*_2_ denotes the weight of CP in the residue at various incubation times, *A* fraction represents the percentage of CP that is instantly or completely degraded in the rumen, including non-protein nitrogen and true protein that escapes from the *in situ* bag due to high solubility or very fine particle size. *B* fraction is considered potentially degraded CP, with the amount degraded at time *t* determined from *kd*. C fraction is the percentage of CP that is completely undegraded and assumed to pass entirely to the small intestine. Therefore, RDP comprises the entire *A* fraction and the portion of *B* actually degraded in the rumen. The RUP includes the portion of *B* that exits the rumen before digestion, and the entire *C* fraction. The sum of RDP and RUP equals 100%. The fractional rate of passage from the rumen (*kp*) was set at a value of 0.05 per hour.

### Modified three-step *in vitro* assay for residual DM and CP determination

The rumen-undegraded residues after incubating in the rumen for 16 h were analyzed using the pepsin and pancreatin digestion steps of the modified three-step *in vitro* procedure described by Gargallo et al [14]. This procedure utilized two types of bags to correct for potential impacts of bag pore size on intestinal digestibility: bags with a pore size of 50 μm (5×10 cm [width×height]; R510; Ankom Technology, USA) and bags with a pore size of 25 μm (5×5 cm [width×depth]; F57; Ankom Technology, USA).

For each bag type, 5 g of sample was measured into Ankom R510 bags, and 1 g of sample into Ankom F57 bags, and both types of bags were incubated in a Daisy II incubator (D200; Ankom Technology, USA) at 39°C for 1 h using a 1 g/L pepsin solution (P7000; Sigma, St. Louis, MO, USA) adjusted to pH 1.9. After incubation, the bags were rinsed with tap water and then incubated again at 39°C for 24 h in a 3 g/L pancreatin solution (P7545; Sigma, USA). The maximum number of bags per jar was limited to 15. Following the 24 h incubation, the liquid was drained from the jars, and the bags were rinsed until the effluent was clear. The bags were then dried in a dry oven at 55°C for 48 h. After drying, the dry weight of the sample and bag was recorded, and the bags were opened to collect the samples for CP analysis as described in the chemical analysis section.

The estimated intestinal dietary protein digestibility (IDP, % of RUP) was determined from the feed residues, representing the intestinal digestibility of RUP. While Gargallo et al [14] recommended incubating feed samples in the rumen for 12 h, this study estimated the IDP of RUP from samples incubated in the rumen for 16 h, based on Calsamigli and Stern [15] that showed no significant difference in protein digestibility with pepsin-pancreatin between 12 and 18 h of rumen incubation. The intestinally absorbable dietary protein (IADP, % of CP) was estimated using the formula: IADP = RUP×IDP. Additionally, the total digestible dietary protein (TDP, % of CP) was estimated as: TDP = RDP+IADP.

### Animal feeding trial

Thirty-six early-fattening Hanwoo steers (initial weight, 480.9±58.6 kg; age 15.9±1.4 months) were allocated into 4 treatments based on weight and age using a randomized complete block design, containing 3 pens per treatment with 3 animals per pen resulting in a total of 12 pens. Based on NIAS [4], the PF treatment had its energy level increased by 10% compared to control, while the PF+SB and PF+SB+HSB had both energy and protein levels increased by 10% relative to control. Two treatment groups were also designed to have differing ratios of RDP to RUP, with the treatments being as follows: control (no supplements; TDN 75%, CP 15%, RDP:RUP = 62:48), PF (TDN 82.5%, CP 15%, RDP:RUP = 62:48), PF+SB (TDN 82.5%, CP 16.5%, RDP:RUP = 62:48), and PF+SB+HSB (TDN 82.5%, CP 16.5%, RDP:RUP = 48:52). The TDN values were calculated using the formula proposed by Conrad [16]: 0.93×CP+0.92×(1+EE–ash–CP–NDF)+0.75×(NDF–ADL)×(1–[ADL]^2/3^/[NDF]^2/3^).

Protected fat (Energy booster 100; Milk Specialties Global, Eden Prairie, MN, USA) consisted of prilled fat based on palm oil with 99.5% free fatty acids, including 2.5% myristic acid (C 12:0), 28.0% palmitic acid (C 16:0), 45.0% stearic acid (C 18:0), 8.3% oleic acid (C 18:1), 1.5% linoleic acid (C 18:2), and 0.1% linolenic acid (C 18:3). Heat-treated soybean meal (SoELAB Pass; FEEDUP, Nonsan, Korea) was processed by circulating batch method under conditions of 90°C to 98°C for 30 to 40 minutes, with its chemical and amino acid compositions presented in Table 1. This analysis was specifically conducted to determine whether there were differences in amino acid composition between soybean meal and heat-treated soybean meal. The experimental diet was prepared as a TMR using a horizontal stationary mixer (SI-2A-350S; Silti, Goyang, Korea). To prevent multifactorial results due to the variety of feed ingredients, the number of the ingredients was minimized when composing the formula, and both the formula and chemical composition of experimental TMR diets are shown in Table 2.

The feeding trial was conducted for a total of 112 days (16 weeks) from July 7, 2022, to November 3, 2022. Body weight (BW) was measured every 4 weeks before morning feeding from the initial to the end of the experiment. The experimental diets were fed twice daily (at 7:00 and 17:00) and were restricted to 10.5 kg per a steer per day (DM basis; assuming 30% moisture) based on NIAS for the fattening period of Hanwoo steers [4]. The amount of residual feeds was measured before the morning feeding at the following day to calculate feed intake. Water and mineral blocks were freely accessible. The FCR was calculated using the formula: DMI (kg)/ADG (kg/d).

### Physiological parameters

Rectal temperature (RT) measurements and blood sampling were conducted every 4 weeks from the initial to the end of the experiment. The RT was measured at 14:00 after blood collection using a thermometer (KD-133; Polygreen Co., Ltd., Berlin, Germany), assuming the time when heat stress peaks. For accurate measurement of RT of the animals, the thermometer was inserted 3 cm deep into the rectum and maintained in contact with the mucosa for 1 minute [17].

From the jugular vein of each steer, 20 mL of blood was collected and stored in non-heparinized vacutainers (10 mL; Becton-Dickinson, Franklin Lakes, NJ, USA) and ethylenediaminetetraacetic acid (EDTA)-treated vacutainers (10 mL; Becton-Dickinson, USA). The collected blood samples were centrifuged at 2,700 g for 15 minutes at 4°C for serum separation, then transferred to 1.5 mL tubes (Eppendorf AG, Hamburg, Germany) and stored at −80°C until further analysis.

Subsequently, the serum samples were analyzed for cortisol using a bovine ELISA test kit (MBS70325; MyBioSource Inc., San Diego, CA, USA). An automatic chemistry analyzer (CHEM 7000i; Fujifilm, Tokyo, Japan) was utilized to measure the levels of glucose, blood urea nitrogen (BUN), and glutamic oxaloacetic transaminase (GOT). Analytical reagents from WAKO (Osaka, Japan) were used for the measurement of non-esterified fatty acids (NEFA).

### Measurement of ambient temperature and relative humidity

The feeding trial was conducted at a commercial Hanwoo farm located at latitude 37.90092507299778 and longitude 126.991961753878. Climate data were recorded every 10 minutes using 4 temperature and relative humidity data loggers (MHT-381SD; Lutron Electronics Inc., Coopersburg, PA, USA) installed inside the pens. The recorded data were collected every 4 weeks, and the temperature-humidity index (THI) was calculated using the formula provided by the NRC [18]: ([1.8×T_db_+32]–[0.55–0.0055×RH]×[1.8×T_db_–26]; T_db_, dry-bulb temperature [°C]; RH, relative humidity [%]) The minimum, maximum, and average values of the ambient temperature, relative humidity, and THI for each period (4 weeks) are presented in Table 3.

### Observation of animal behaviors

Animal behaviors were observed through closed circuit television (CCTV, DS-2CE16D0T-IRP 3.6 mm; HIKVISION, Seoul, Korea), with one pen containing three steers per treatment group being selected for continuous recording over a period of 3 days. To ensure full visibility of the pen without any blind spots, two CCTV cameras were diagonally installed at the front and back of each pen.

Behavioral measurements were categorized into 7 types: lying, total standing, walking, eating, drinking, rumination during standing, and rumination during lying, conducted by the previous research [9]. Rumination included both lying and standing, while total standing included walking, eating, rumination, and drinking. The behavioral analysis was based on the heat stress indicators for fattening Hanwoo steers provided by NIAS [4], comparing the differences between hot season and post-hot season. The analysis was measured on days when moderate (THI 82 to 84, 1st) and comfort (THI 68 to 75, 3rd) levels lasted for more than 5 days, respectively.

### Statistical analysis

All experimental data were analyzed using the MIXED procedure of SAS 9.4 (SAS Inst. Inc., Cary, NC, USA). The statistical model in data of *in situ* degradability included feed ingredient as a fixed variable and animal as a random variable. *In vitro* data included feed ingredient as a fixed variable. Data of THI included period as a fixed variable and a logger as a random variable. Data of *in vivo* included dietary treatments as fixed variables and a pen as a random variable. The experimental unit was a pen including three steers. Data of behaviors included period as fixed variables and animal as fixed variables.

Data of physiological parameters were analyzed to a repeated measurement over time using the procedure of Littell et al [19]. Dietary treatments, days, and their interaction were included in the statistical models as fixed variables, and pens were treated as random variables. The compound symmetry was used to ensure that measures were always qualified for the same variance and that all pairs of measures for the same animal were equally correlated. Least squares means of each treatment was calculated. The difference of means was tested using the PDIFF option with Tukey’s adjustment. An alpha level of 0.05 was used to assess the statistical significance.

## RESULTS AND DISCUSSION

### *In situ* ruminal DM and CP degradability of feed ingredients for experimental TMR diet

**Figure 1:**
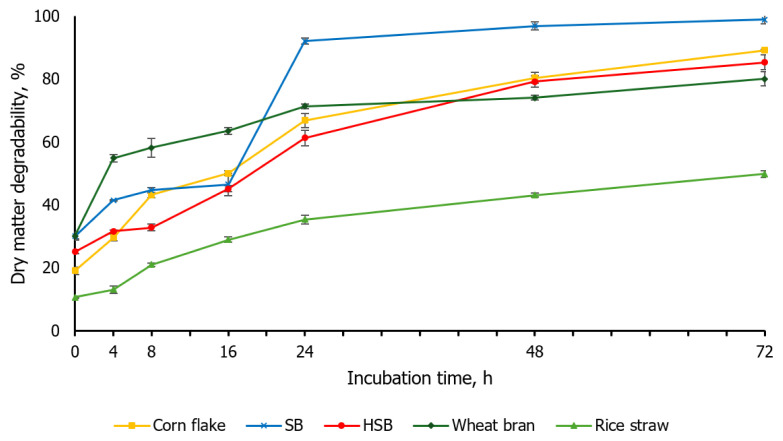
*In situ* dry matter degradability of feed ingredients on incubating from 0 to 72 h (soybean meal, SB; heat-treated soybean meal, HSB). Error bars indicate standard error.

The *in situ* ruminal DM degradability of the feed ingredients used in the experimental TMR diets for this *in vivo* trial is shown in Figure 1. For accurate comparison, the SB used in this *in situ* measurement and *in vivo* trial was the same feed ingredient prior to heat treatment of HSB. At 0 h of incubation, the degradability of raw SB and wheat bran exceeded 30%, while that of HSB was 25.14% (p<0.05). Lee et al [20] also reported that the DM degradability of soybean meal at 0 h was more than 30% and Pan et al [21] reported that the degradability of wheat bran at 0 h was close to 50%. The degradability of corn flake increased continuously from 19.05% at 0 h to 80.42% at 48 h. The degradability of SB slightly rose from 41.47% at 4 h to 46.51% at 16 h, then exponentially increased to 92.03% at 24 h. Conversely, the degradability of HSB gradually increased to 31.67% at 4 h, 32.84% at 8 h, 45.17% at 16 h, 61.31% at 24 h, and 79.24% at 48 h, significantly lower than the 96.92% at 48 h for raw SB (p<0.05). The DM degradability of wheat bran linearly increased to 43.12% at 48 h from 10.65% at 0 h. The final DM degradability (72 h) for corn flake, SB, HSB, wheat bran, and rice straw were 89.15%, 98.99%, 85.36%, 80.13%, and 49.82%, respectively. Assuming an outflow rate of 4% (Table 4), the ED was the highest for SB (70.98%), followed by wheat bran (63.53%), corn flake (56.95%), HSB (53.26%), and rice straw (29.60%) (p<0.05).

**Figure 2:**
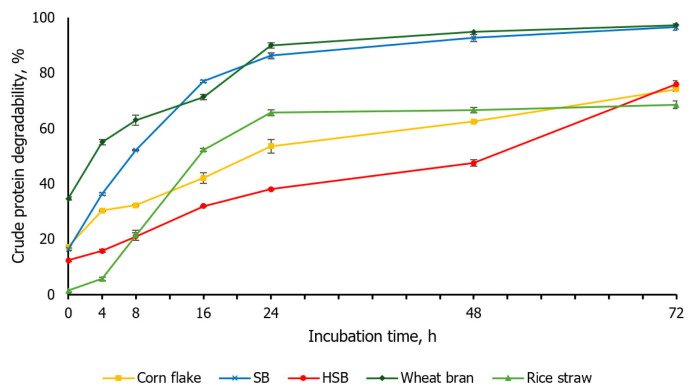
*In situ* crude protein degradability of feed ingredients on incubating from 0 to 72 h (soybean meal, SB; heat-treated soybean meal, HSB). Error bars indicate standard error.

The *in situ* ruminal CP degradability of the feed ingredients is presented in Figure 2. The CP degradability of corn flake at 0 h was 17.25%, showing continuous increase to 74.16% at 72 h. The raw SB for the degradability at 0 h was 16.32%, exponentially increase to 77.05% at 16 h and continuously increase to 96.63% at 72 h. Maxin et al [22] reported CP degradability of SB at 0 and 16 h was 23% and 78%, respectively. Compared to this study, the degradability at 0 h was higher, but the degradability at 16 h was similar. The degradability of HSB at 0 h (12.36%) was lower compared to raw SB (p< 0.05), with gradual increases to 47.48% at 48 h. Therefore, considering that the retention time of SB in the rumen is less than 48 h and mostly degraded within 24 h [20], the 48 h of degradability was 45% units lower than raw SB. The ruminal CP degradability of both initial and final times for wheat bran were the highest among the feed ingredients in this *in situ* experiment (p<0.05), reaching 34.67% and 97.31%, respectively. Pan et al [21] reported that the CP degradability of wheat bran was more than 30% at 0 h and 83% at 16 h. Compared to this study, the degradability at 0 h was similar, but the degradability at 16 h (71.41%) was approximately 12% unit different. After 24 h, both studies showed the degradability of over 90%. The CP degradability for rice straw was from 1.38% at 0 h to 68.56% at 72 h.

The RUP (% of CP) values were highest for HSB (70.03%), followed by corn flake (58.49%), rice straw (55.79%), SB (33.44%), and wheat bran (27.41%). However, considering the CP content of each feed ingredient, the actual contribution of true RUP (% of DM) to the total diet was 37.10% for HSB, 17.57% for SB, 4.70% for corn flake, 4.42% for wheat bran, and 2.23% for rice straw, thus the proportion of corn flake, wheat bran and rice straw being significantly lower than those of SB and HSB. The NRC Beef Cattle [3] model estimated RUP for corn flake, SB, and wheat bran as 70%, 29%, and 36%, respectively, while the NRC Dairy Cattle [23] estimated them as 69%, 63%, and 18%, respectively. This difference is due to the fact that NRC Beef Cattle [3] presented RUP values for feed ingredients calculated by uniformly applying a 5% outflow rate (*kp*) using the fraction system (A, B, C, *kd*) to the *in situ* rumen CP degradability. Contrarily, in the NRC Dairy Cattle [23], the fraction system was maintained but the outflow rate for each feedstuff was calculated to derive the values. The calculation of *in situ* CP degradability in this experiment was based on NRC Beef Cattle [3], showing similar values. In addition, data on RUP for rice straw were scarce, and the protein content of rice straw is considered to have a minimal effect on performance.

### Modified three-step *in vitro* protein digestibility of feed ingredients

The *in vitro* digestibility of RUP in the small intestine for the feed ingredients is presented in Table 5. The IDP (% of RUP) for corn flake, SB, and HSB were 92.72%, 94.35%, and 96.61% respectively, indicating similar values. The IDP of wheat bran was 71.91%, which was lower than those of corn flake, SB, and HSB but higher than that of rice straw (52.31%) (p<0.05).

The IADP (% of CP) was the highest for HSB (67.65%), followed by corn flake (54.23%), soybean meal (31.55%), rice straw (29.19%), and wheat bran (19.71%) in descending order (p<0.05). Considering the CP content of each feed ingredient, the actual contribution of the true IADP (% of DM) to the total diet for corn flake, SB, HSB, wheat bran, and rice straw was 4.4%, 16.6%, 35.8%, 3.2%, and 1.2%, respectively. Thus, the difference between SB (16.6%) and HSB (35.8%) was approximately 19% units.

The TDP (% of CP) was the highest for SB (98.11), showing a slightly lower value for corn flake (95.74%) (p<0.05). However, HSB (97.62%) showed a similar value to SB, which is attributed to its low RDP but increased IADP after heat treatment. Therefore, HSB is considered as an appropriate feed ingredient to increase CP content when considering RUP. Wheat bran (92.30) showed lower values compared to corn flake, SB, and HSB but was higher than rice straw (73.40%) (p<0.05).

Several studies [24,25] estimating IDP for protein sources using the same also reported high digestibility over 97% for SB. According to the NRC [23], the IADP for corn flake, SB, and wheat bran were 90%, 93%, and 69%, respectively, being similar to the results of this experiment. Meanwhile, when formulating the actual feed formula, it is important to consider not only the RDP and RUP ratios but also the intestinal digestibility of each feed ingredient.

### Animal performance

During the whole experimental period (16 weeks), the average, minimum, and maximum values of ambient temperature, relative humidity, and THI on every 4 weeks are presented in Table 3. From the 1st to the 4th period, the average temperature, humidity, and THI sequentially decreased as the period progressed (p<0.05). Average temperature, humidity, and THI during the hottest 1st period were 29.1°C, 72.3%, and 82.9, respectively, which correspond to moderate (THI 82 to 84) level of heat stress according to the THI chart for fattening Hanwoo steers based on NIAS [4]. In the 2nd period, these were 26.9°C, 62.5%, and 76.9, respectively, indicating mild (THI 76 to 81) heat stress level. The 3rd period following the hot period (1st and 2nd period), had average temperature, humidity, and THI of 23.5°C, 65.3%, and 70.9, respectively, and the 4th period had 19.8°C, 67.9%, and 65.8, with both periods experiencing conditions under comfort (THI below 75) level.

The BW, DMI, ADG, and FCR of fattening Hanwoo steers measured every 4 weeks from the initial to the end of the experiment for control, PF, PF+SB, and PF+SB+HSB are presented in Table 6. The initial weights for the control, PF, PF+SB, and PF+SB+HSB were 479.1, 492.6, 482.5, and 485.0 kg respectively, showing no significant difference between treatments. The final weights for control, PF, PF+SB, and PF+SB+HSB were 589.2, 617.6, 615.5, and 624.6 kg respectively, showing weight gains of 110.1, 125.0, 133.0, and 139.6 kg for each treatment, respectively.

According to the NIAS [4], to prevent sudden decreases or refusal of feed intake during the late-fattening period, the amount of recommended daily TMR diet per an early-fattening Hanwoo steer is 10.5 kg (DM basis), which was adhered to in this experiment through restricted feeding. No significant difference in DMI was observed among treatments during the whole experimental period. However, during the 1st period, the DMI for control, PF, PF+SB, and PF+SB+HSB was 6.97, 6.99, 6.95, and 6.95 kg/d, respectively, which decreased by 34% compared to the recommended daily TMR intake (10.5 kg/d; DM basis). Previous research on feeding additional TDN and CP to Hanwoo steers during the late-fattening period reported a similar reduction of 33% in DMI at severe conditions (THI above 87.0). Thus, despite being the moderate level in this study, a similar reduction in DMI was observed as in the severe conditions. This could be due to the narrow range of moderate (THI 82 to 84) level, potentially exposing the animals to severe conditions [6] and the relatively higher roughage ratio in the TMR during the early-fattening period compared to the late-fattening (25% vs 12%, respectively), leading to higher heat production from ruminal microbial fermentation and consequently reduced feed intake to mitigate the heat [26]. Similar to the results observed in the chamber study with early-fattening Hanwoo steers by Woo et al [6], it was interpreted that in Severe conditions, the DMI of forage was lower than that of formula feed, which was closely related to heat production in the rumen. During the 2nd period, with Mild level (THI 76.9), the DMI was 8.78, 8.86, 8.86, and 8.83 kg/d for control, PF, PF+SB, and PF+SB+HSB, respectively, which is 19% lower than the recommended daily TMR intake. As the temperature moved to Comfort level in the 3rd period, DMI increased to 9.98, 9.99, 10.00, and 9.98 kg/d, respectively, and in the 4th period, it reached 10.02 kg/d across all treatments, indicating nearly complete recovery of feed intake.

During the hottest 1st period, the ADG for Control, PF, PF+SB, and PF+SB+HSB was 0.57, 0.68, 0.70, and 0.79 kg/d, respectively (p<0.05). Thus, PF+SB+HSB, the treatment with TDN and CP levels increased by 10% each considering RUP, showed a 39% improvement in ADG compared to control (p<0.05). No difference was observed between PF and PF+ SB, and these two treatments showed intermediates between control and PF+SB+HSB. The ADG during the 2nd period was higher for PF+SB (1.16 kg/d) and PF+SB+HSB (1.14) compared to control (0.95) (p<0.05). In the 3rd period, PF+ SB+HSB (1.51 kg/d) showed no significant difference from PF+SB (1.43) but was approximately 10% higher than PF (1.37) and 24% higher than control (1.22) (p<0.05). The 4th period showed a similar trend to the 3rd period, and control, PF, PF+SB, and PF+SB+HSB were 1.19, 1.33, 1.42, and 1.48 kg/d, respectively. Overall, the ADG improved during the 1st and 2nd period for treatments with protein supplementation, especially noticeable in the treatment with HSB. Moreover, during the recovery period (3rd and 4th period) after the hot season, treatments with HSB showed improved ADG compared to both control and PF. The ADG during the whole experimental period increased significantly in the order of PF+SB+HSB (1.23), PF+SB (1.18), PF (1.11), control (0.98 kg/d) (p<0.05), suggesting the cumulative effects of the entire feeding experiment over 16 weeks were expressed.

A study by Kang et al [27] showed no significant difference in ADG, probably due to the low level of energy increase when PF was used to increase the TDN by 3% in growing Hanwoo steers. Another study on energy levels by Jo et al [7] conducted an experiment with Hanwoo calves under comfort (THI 70 to 73) or severe (THI 89 to 91) conditions in a controlled temperature and humidity chamber, fixing the CP at 17.5% and tested the TDN levels of 70%, 73%, and 75% using corn (2×3 factorial arrangement). This study reported a difference in ADG between Comfort and Severe, but no difference between TDN levels under the same heat stress conditions. These results suggested that energy additions of less than 5% do not influence ADG. On the other hand, a protein level study by Kim et al [17] fixed the TDN at 73% in the diet of Hanwoo calves and used SB to conduct CP levels of 12.5%, 15%, and 17.5% (3×3 factorial) with 3 THI levels in a controlled temperature and humidity chamber. This study reported that improvement in ADG was observed with increasing level of CP as heat stress increased from mild (THI 70 to 73) to moderate (THI 74 to 76) and to severe (THI 89 to 91).

The above studies in Hanwoo [7,17,27] have investigated the effects of increasing either energy or protein levels under heat stress conditions. Previous research [9] demonstrated that while treatments increasing TDN levels by 10% showed a positive impact on ADG in late-fattening Hanwoo steers during the hot season, treatments that increased CP levels by 10% without considering RUP did not show a clear difference compared to other treatments. This might be due to excess ammonia production in the rumen from the simple addition of SB to increase CP levels, leading to urea excretion [4]. In conclusion, compared to the ADG of Control during the whole period, PF improved by 13%, PF+SB by 20%, and PF+SB+HSB by 26%, although the absolute differences were not large.

During the 1st and 2nd period, the FCR improved in PF+SB+HSB compared to control (p<0.05). In the 3rd and 4th period, the FCR among control, PF, PF+SB, and PF+SB+ HSB showed similar changes in ADG (p<0.05). During the whole period, the FCR sequentially decreased for control (9.10), PF (8.09), PF+SB (7.61), PF+SB+HSB (7.27) (p<0.05). Therefore, this study showed that feeding TMR diets with an increased TDN level using PF and CP level considering RUP by 10% each during the early-fattening period of Hanwoo steers can prevent the decline in performance under heat stress and positively affect performance recovery after the hot season.

### Physiological parameters

**Figure 3:**
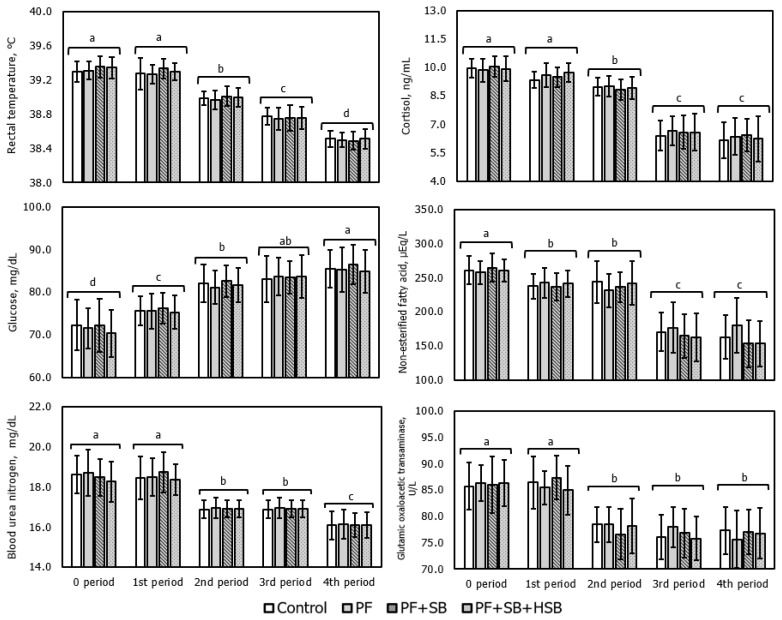
Changes in physiological parameters according to experimental periods in early-fattening period of Hanwoo steers under heat stress conditions. Values are least squares means±standard error for each treatment. a–d The means with different letters differ (p<0.05) by experimental periods, not by treatments. No significant differences were within each period among treatments.

The results from increasing TDN and CP levels by 10% each using PF, SB, and HSB in Hanwoo steers during the early-fattening period under heat stress on physiological parameters are presented in Table 7 and Figure 3. During the whole period, there was no significant difference in all physiological parameters among treatments. However, when comparing each period, average RT for the 0, 1st, 2nd, 3rd, and 4th period were 39.33°C, 39.30°C, 38.99°C, 38.76°C, and 38.70°C, respectively, showing differences, albeit not large, with high significance (p<0.001). Therefore, the RT during Moderate of 0 and 1st period was the highest, followed by the 2nd period (mild) being higher than the 3rd and 4th period (comfort) (p<0.05; Figure 2). In addition, the RT of 3rd period was higher than that of 4th period (p<0.05). An increase in RT in ruminants indicates an increase in endogenous heat production [28]. According to Woo et al [6], the RT of Hanwoo steers during early-fattening period in a chamber with controlled temperature and humidity was reported as 37.39°C, 37.80°C, 38.65°C, and 39.20°C for comfort (THI 73 to 75), mild (THI 77 to 79), moderate (THI 82 to 84), and severe (THI 85 to 86) levels, respectively (p<0.05). Moreover, previous research [9] showed that the average RT under ambient temperature conditions for Hanwoo steers in the late-fattening period increased in the order of 4th (comfort; 38.48°C), 3rd (comfort; 38.46°C), 2nd (moderate; 38.76°C), 1st (severe; 38.85°C), and 0 period (severe; 38.95°C) (p<0.05). These findings support the observation that the extent of heat stress during the 1st period in this experiment was similar to severe level, leading to a similar decrease in DMI.

When compared among periods, average serum cortisol concentrations were 9.95, 9.54, 8.94, 6.56, and 6.31 ng/mL for the 0, 1st, 2nd, 3rd, and 4th period, respectively (p<0.001). Serum cortisol concentrations were the highest during the moderate stage of 0 and 1st period and were higher in the 2nd (mild) than in the 3rd and 4th period (comfort) (p<0.05; Figure 2). Similar to the results of this study, previous research [9] found cortisol concentrations of 8.86, 8.44, 8.41, 7.07, and 7.07 ng/mL for the 0, 1st, 2nd, 3rd, and 4th period, respectively, with significant differences over time. When comparing the results of this study with previous research [9], the concentrations were higher than Severe even though the heat stress was at moderate. Similar to RT, these physiological parameters probably influenced DMI. Another study [29] observed an increase in cortisol concentrations of Hanwoo steers during early-fattening period under ambient conditions to at THI 80 to 87 (9.87 ng/mL) compared to 1.91 and 5.13 ng/mL at THI 64 to 71 and THI 72 to 79, respectively. However, this study suggested that cortisol concentrations could exceed 9.5 ng/mL at THI 82.9 in Moderate conditions. The increase in cortisol concentrations in ruminants under heat stress is due to adrenal cortex secretion in response to heat stress [30]. Hence, changes in RT and serum cortisol concentrations indicate that animals of this feeding trial undergo heat stress during the 1st and 2nd period.

Average serum glucose concentrations for the 0, 1st, 2nd, 3rd, and 4th period were 71.60, 75.72, 81.87, 83.49, and 85.51 mg/dL, respectively (p<0.001), with the lowest concentration during the 0 period and increasing through the 1st, 2nd, and 4th period (p<0.05; Figure 2). However, the 3rd period was not significantly different from either the 2nd or 4th period. Previous research [6] also reported a decrease in serum glucose with increasing THI. The primary reasons for this result were changes in glucose production due to decreased feed intake and increased insulin concentration in the body [8]. Furthermore, PF may not have a direct effect on serum glucose concentrations because they do not interfere with rumen fermentation processes and can be absorbed by the small intestine to provide more energy for the ruminant [9,31].

Average serum NEFA concentrations for the 0, 1st, 2nd, 3rd, and 4th period were 261.0, 239.5, 238.2, 172.8, and 158.6 μEq/L, respectively (p<0.001), with the highest concentration during the 0 period (p<0.05; Figure 2). Several studies [6,7,9,17] have reported a decrease in blood glucose and an increase in NEFA concentrations under heat stress. The increase in NEFA concentration due to decreased blood glucose is related to subcutaneous lipid breakdown, acting as an alternative energy source [32].

Average serum BUN concentrations the 0, 1st, 2nd, 3rd, and 4th period were 18.52, 18.51, 16.92, 16.92, and 16.10 mg/dL for, respectively (p<0.001), with the highest concentrations during the 0 and 1st period and higher concentrations in the 2nd and 3rd period compared to the 4th period (p< 0.05; Figure 2). Similar to the previous research [9], concentrations of BUN were not changed by nutritional differences among treatments. However, BUN concentrations were higher during severe (THI 87.0) and moderate (THI 82.8) levels compared to comfort levels (THI 71.4 and 68.1). This could be due to increased blood BUN during heat stress, resulting from inefficient incorporation of ruminal ammonia into microbial protein or deamination of amino acids mobilized from skeletal muscle in the liver [8]. Secondly, heat stress may lead to inefficient metabolism of rumen protein and amino acid imbalance, reducing the absorptive function of the rumen epithelium and leading to an accumulation of BUN in the blood [33].

Average serum GOT concentrations for the 0, 1st, 2nd, 3rd, and 4th period were 86.12, 86.08, 77.96, 76.71, and 76.70 U/L, respectively (p<0.001), with higher concentrations during the 0 and 1st period compared to the rest of periods (p<0.05; Figure 2). Serum GOT concentration is a marker for liver cell damage and can indicate the extent of liver function impairment due to heat stress [8]. According to NIAS [4], serum GOT concentrations during severe (THI 85 and above), moderate (THI 82 to 84), and mild (THI 82 to 84) levels of heat stress were reported as 85 and above, 79 to 85, and 75 to 78 U/L, respectively. The GOT concentrations of this study were indicative of severe levels suggested by NIAS [4]. In conclusion, physiological indicators such as RT, serum cortisol, glucose, NEFA, BUN, and GOT concentrations did not vary among treatments based on TDN and CP levels in the diets but showed significant differences over period (p<0.001), making them appropriate parameters for determining whether Hanwoo steers have experienced heat stress. In addition, these results imply that the stages of the Hanwoo THI chart presented by NIAS [4] are well differentiated.

### Animal behaviors

Animal behavioral measurements with hot and post-hot season in early-fattening Hanwoo steers fed a 10% increase in TDN and CP levels are presented in Table 8. The primary aim of measuring animal behaviors was not to focus on behavioral changes due to variations in nutritional levels across treatments but to compare changes in behaviors under heat stress conditions. No significant differences were observed in any behavioral measurement due to differences in diets among treatments. Consequently, significant changes in behaviors between the 1st (moderate; July) and 3rd (comfort; September) period were identified.

Lying decreased by approximately 24% in the 1st period compared to the 3rd, while total standing increased by 50% (p<0.05). Additionally, walking decreased by 48% in the 1st period compared to the 3rd (p<0.05). Previous research [9] also found that Lying decreased by 43% from comfort (THI 71) to severe (THI 87), total standing increased by 48%, and walking decreased by 62%. Another study [29] reported that lying in Hanwoo steers during the early-fattening period decreased by 25% from comfort (THI 64 to 71) to severe (THI 80 to 87), while standing increased by 11%. It is known that ruminants try to minimize contact with the ground and maximize body surface area to reduce body temperature under heat stress [3].

Eating decreased by 40% in the 1st period compared to the 3rd, while drinking increased by 43% (p<0.05). This was interpreted as a result of a 30% decrease in DMI during the 1st period compared to the 3rd period. Previous research [9] also reported a decrease in DMI by 36% from comfort to severe, resulting in a decrease in eating by 38% and an increase in drinking by 54%. The decrease in eating time and increase in drinking time are attributed to sweating and panting to regulate body temperature under heat stress. This respiratory and thermoregulatory water loss can lead to an increase in water intake, which in turn may also influence the decrease in DMI [3].

Rumination during standing increased by 38% in the 1st period compared to the 3rd, while Rumination during lying decreased by 32% (p<0.05). This was because lying decreased by 24%, and total standing increased by 50%. Previous research [9] showed that rumination during standing increased by 53% from comfort to severe, while rumination during lying decreased by 33%. Total rumination time was relatively lower during the hot season compared to the post-hot season, primarily due to the effect of decreased feed intake under heat stress, leading to reduced rumen motility and increased retention time of feeds in the rumen [8]. Therefore, changes in rumination during standing and rumination during lying during the hot season of this experiment were considered as appropriate indicators for assessing the degree of heat stress in ruminants. Furthermore, although the extent of changes in behavioral measurements varied between this study and the previous research [9] due to differences in experimental conditions (level of heat stress, weight, age, feed intake, forage-to-concentrate ratio), the trends in changes were similar.

## CONCLUSION

Increasing dietary TDN by 10% using PF and adjusting CP levels considering RUP can effectively mitigate performance reduction in early-fattening Hanwoo steers under heat stress conditions. The diet not only improved ADG during heat stress but also helped performance recovery following the stress period. This is the first study demonstrating that RUP can improve performance of beef cattle under heat stress conditions.

## Figures and Tables

**Table 1:** Chemical composition of feed ingredients for experimental total mixed ration diet (DM basis)

Items (%)	Corn flake	SB	HSB	Wheat bran	Rice straw
DM	86.68	88.88	91.19	88.45	75.39
CP	8.03	52.55	52.98	16.12	3.99
EE	3.23	0.69	0.35	2.39	0.73
Ash	1.72	7.70	7.96	5.46	8.24
CF	1.32	5.94	6.01	11.21	36.92
NDF	13.88	21.80	25.74	44.98	66.63
ADF	3.06	5.75	5.87	11.19	34.52
ADL	1.85	1.24	1.49	2.53	4.22
NDICP	1.28	1.51	1.57	2.75	1.55
ADICP	0.61	1.05	1.12	1.61	0.63
TDN	87.36	79.15	77.32	75.13	63.20
Indispensable amino acids					
Arg	0.39	3.68	3.56	1.02	0.21
His	0.24	1.36	1.33	0.42	0.05
Ile	0.28	2.28	2.32	0.46	0.15
Leu	0.99	3.94	4.01	0.96	0.29
Lys	0.24	3.17	2.85	0.67	0.17
Met	0.15	0.71	0.77	0.23	0.07
Phe	0.40	2.64	2.72	0.62	0.19
Thr	0.31	2.09	2.17	0.53	0.19
Val	0.39	2.42	2.48	0.72	0.24
Dispensable amino acids					
Ala	0.61	2.26	2.32	0.78	0.27
Asp	0.54	5.87	6.03	1.07	0.37
Cys	0.18	0.77	0.80	0.35	0.09
Glu	1.48	9.27	9.46	2.89	0.46
Gly	0.32	2.22	2.24	0.84	0.21
Pro	0.73	2.72	2.76	1.01	0.27
Ser	0.40	2.77	2.79	0.68	0.19
Tyr	0.30	1.71	1.70	0.36	0.09

DM, dry matter; SB, soybean meal; HSB, heat-treated soybean meal; CP, crude protein; EE, ether extract; CF, crude fiber; NDF, neutral detergent-insoluble fiber; ADF, acid detergent-insoluble fiber; ADL, acid detergent lignin; NDICP, neutral detergent-insoluble crude protein; ADICP, acid detergent-insoluble crude protein; TDN, total digestible nutrient.

**Table 2:** Formula and chemical composition of experimental total mixed ration diets for Hanwoo steers of early-fattening period

Items (%)	Treatments^1)^			

	Control	PF	PF+SB	PF+SB+HSB
Formula (as-fed basis)				
Protected fat	-	3.01	3.01	3.01
SB	10.09	11.71	14.98	8.06
HSB	-	-	-	5.63
Wheat bran	18.01	15.01	11.01	16.80
Corn flake	27.00	25.05	25.74	21.11
Rice straw	22.22	22.18	22.18	22.18
Water	20.60	20.96	21.00	21.13
Vitamin and mineral premix	0.28	0.28	0.28	0.28
Limestone	1.00	1.00	1.00	1.00
Salt	0.40	0.40	0.40	0.40
Sodium bicarbonate	0.40	0.40	0.40	0.40
Total	100	100	100	100
Chemical composition (DM basis)				
Dry matter	67.71	67.26	67.92	67.35
Crude protein	14.85	15.12	16.45	16.57
Ether extract	4.27	10.34	11.18	10.82
Ash	8.13	7.82	8.05	8.17
Neutral detergent-insoluble fiber	36.55	36.72	36.88	35.47
Acid detergent-insoluble fiber	14.59	13.68	14.02	13.98
Acid detergent lignin	3.21	2.44	2.56	2.53
Total digestible nutrients	75.04	82.18	82.51	82.43

PF, protected fat; SB, soybean meal; HSB, heat-treated soybean meal; RDP, rumen degradable protein; RUP, rumen undegradable protein; CP, crude protein; TDN, total digestible nutrient.

1) Control, no supplements, RDP:RUP = 62:38; PF, 10% more TDN with PF, RDP:RUP = 62:38; PF+SB, 10% more TDN and CP with PF and SB, RDP:RUP = 62:38; PF+SB+HSB, 10% more TDN and CP with PF, SB, and HSB, RDP:RUP = 48:52.

**Table 3:** Mean, maximum, and minimum values of ambient temperature, relative humidity, and THI during the experimental period

Items	Experimental period^1)^				SEM	p-value

	1st	2nd	3rd	4th		
Ambient temperature (°C)						
Mean	29.1^a^	26.9^b^	23.5^c^	19.8^d^	0.76	<0.001
Maximum	39.9^a^	38.8^a^	34.9^b^	32.9^c^	1.20	<0.001
Minimum	23.6^a^	19.6^b^	12.6^c^	8.4^d^	0.95	<0.001
Relative humidity (%)						
Mean	72.3^a^	62.5^d^	65.3^c^	67.9^b^	2.35	<0.001
Maximum	82.7^b^	82.0^c^	81.2^d^	84.3^a^	0.93	<0.001
Minimum	36.7^b^	40.7^a^	33.1^c^	29.8^d^	2.88	<0.001
THI^2)^						
Mean	82.9^a^	76.9^b^	70.9^c^	65.8^d^	2.59	<0.001
Maximum	91.2^a^	88.2^b^	86.9^c^	84.1^d^	0.65	<0.001
Minimum	71.8^a^	66.0^b^	55.1^c^	48.3^d^	2.92	<0.001

SEM, standard error of the means; THI, temperature-humidity index.

1) 1st period (0 to 4 week), 220707–220811; 2nd period (5 to 8 week), 220812–220908; 3rd period (9 to 12 week), 220909–221006; 4th period (13 to 16 week), 221007–221103.

2) THI = (1.8×ambient temperature+3^2)^–(0.55–0.0055×relative humidity)×(1.8×ambient temperature–26) [18].

a–d Means within a row without a common superscript letter differ (p<0.05).

**Table 4:** Rumen degradation characteristics of dry matter and crude protein of feed ingredients for experimental total mixed ration diet

Items (%)	Corn flake	SB	HSB	Wheat bran	Rice straw	SEM	p-value
Degradation characteristics of DM and ED							
a^1)^	15.73^b^	12.32^b^	14.74^b^	46.32^a^	10.47^b^	0.043	0.009
b^2)^	71.33^a^	86.66^a^	71.16^a^	33.81^b^	39.35^b^	0.041	0.002
c^3)^	0.055^ab^	0.084^a^	0.047^ab^	0.042^b^	0.038^b^	0.007	0.025
a+b^4)^	87.06^b^	98.99^a^	85.90^b^	80.13^c^	49.82^d^	0.009	<0.001
ED02^5)^	67.98^b^	82.24^a^	64.72^c^	69.07^b^	36.21^d^	0.004	<0.001
ED04^5)^	56.95^c^	70.98^a^	53.26^d^	63.53^b^	29.60^e^	0.003	<0.001
ED06^5)^	49.77^c^	62.87^a^	46.07^d^	60.17^b^	25.69^e^	0.005	<0.001
Degradation characteristics of CP, ED, RDP, and RUP							
a	20.13^bc^	27.83^ab^	14.03^cd^	37.83^a^	3.23^d^	0.023	<0.001
b	53.15^b^	68.79^a^	61.98^ab^	59.48^ab^	65.32^a^	0.024	0.025
c	0.034^c^	0.064^b^	0.017^c^	0.070^ab^	0.084^a^	0.003	<0.001
a+b	73.28^bc^	96.63^a^	76.01^b^	97.31^a^	68.56^c^	0.010	<0.001
ED02	53.47^c^	80.33^b^	42.76^d^	84.12^a^	56.01^c^	0.005	<0.001
RDP^6)^	41.51^c^	66.56^b^	29.97^d^	72.59^a^	44.21^c^	0.482	<0.001
RUP^7)^	58.49^b^	33.44^c^	70.03^a^	27.41^d^	55.79^b^	0.482	<0.001

SB, soybean meal; HSB, heat-treated soybean meal; SEM, standard error of the means; DM, dry matter; ED, effective degradability; CP, crude protein; RDP, rumen degradable protein; RUP, rumen undegradable protein.

1) a, rapidly degradable fraction (%).

2) b, insoluble fraction but degraded over time in rumen (%).

3) c, constant for b fraction in the exponential equation (fraction/h).

4) a+b, potentially degradable fraction (%).

5) ED (%), effective degradability calculated with outflow rates of 2% 4% and 6%, t = 72 h.

6) RDP (% of CP) = (a+b×(kd/(kd+kp)))×100.

7) RUP (% of CP) = (b×(kp/(kd+kp))+c)×100; kd, rate of degradation of b fraction; kp, rate of passage from the rumen (= 0.0^5)^; c: 100–(a+b) [3].

a–e Means within a row without a common superscript letter differ at p<0.05.

**Table 5:** *In vitro* protein digestibility of feed ingredients after a 16 h ruminal incubation

Items^1)^	Corn flake	SB	HSB	Wheat bran	Rice straw	SEM	p-value
IDP	92.72^a^	94.35^a^	96.61^a^	71.91^b^	52.31^c^	1.033	<0.001
IADP	54.23^b^	31.55^c^	67.65^a^	19.71^e^	29.19^d^	0.459	<0.001
TDP	95.74^b^	98.11^a^	97.62^ab^	92.30^c^	73.40^d^	0.459	<0.001

SB, soybean meal; HSB, heat-treated soybean meal; SEM, standard error of the means; RUP, rumen undegradable protein.

1) IDP (% of RUP), estimated intestinal dietary protein digestibility [14]; IADP (% of CP), intestinally absorbable dietary protein (RUP×IDP); TDP (% of CP), total digestible dietary protein (RDP+IADP).

a–e Means within a row without a common superscript letter differ at p<0.05.

**Table 6:** Effects of supplemental feeding protected fat, soybean meal, and heat-treated soybean meal on growth performance of Hanwoo steers in early-fattening period under heat stress conditions

Items^2)^		Treatments^1)^				SEM	p-value

		Control	PF	PF+SB	PF+SB+HSB		
BW (kg)	0 wk	479.1	492.6	482.5	485.0	20.35	0.971
	4th wk	495.0	512.4	503.1	508.8	20.11	0.934
	8th wk	521.6	542.1	535.7	540.7	20.25	0.886
	12th wk	555.9	580.5	575.9	583.1	20.33	0.777
	16th wk	589.2	617.6	615.5	624.6	20.21	0.628
DMI (kg/d)	1st period	6.97	6.99	6.95	6.95	0.022	0.518
	2nd period	8.78	8.86	8.86	8.83	0.032	0.291
	3rd period	9.98	9.99	10.00	9.98	0.006	0.232
	4th period	10.02	10.02	10.02	10.02	0.001	0.468
	Whole period	8.94	8.96	8.96	8.95	0.012	0.474
ADG (kg/d)	1st period	0.57^b^	0.68^ab^	0.70^ab^	0.79^a^	0.035	<0.001
	2nd period	0.95^b^	1.06^ab^	1.16^a^	1.14^a^	0.031	<0.001
	3rd period	1.22^c^	1.37^b^	1.43^ab^	1.51^a^	0.033	<0.001
	4th period	1.19^c^	1.33^b^	1.42^ab^	1.48^a^	0.029	<0.001
	Whole period	0.98^d^	1.11^c^	1.18^b^	1.23^a^	0.011	<0.001
FCR (F/G)	1st period	12.30^a^	10.28^ab^	9.95^ab^	8.78^b^	0.628	0.003
	2nd period	9.27^a^	8.36^ab^	7.61^b^	7.74^b^	0.313	0.001
	3rd period	8.15^a^	7.29^b^	6.97^bc^	6.60^c^	0.183	<0.001
	4th period	8.41^a^	7.55^b^	7.08^bc^	6.76^c^	0.182	<0.001
	Whole period	9.10^a^	8.09^b^	7.61^c^	7.27^d^	0.086	<0.001

PF, protected fat; SB, soybean meal; HSB, heat-treated soybean meal; SEM, standard error of the means; BW, body weight; DMI, dry matter intake; ADG, average daily gain; FCR, feed conversion ratio; RDP, rumen degradable protein; RUP, rumen undegradable protein; TDN, total digestible nutrient; CP, crude protein.

1) Control, no supplements, RDP:RUP = 62:38; PF, 10% more TDN with PF, RDP:RUP = 62:38; PF+SB, 10% more TDN and CP with PF and SB, RDP:RUP = 62:38; PF+SB+HSB, 10% more TDN and CP with protected fat, SB, and HSB, RDP:RUP = 48:52.

2) 1st period (0 to 4 wk), 220707–220811; 2nd period (5 to 8 wk), 220812–220908; 3rd period (9 to 12 wk), 220909–221006; 4th period (13 to 16 wk), 221007–221103.

a–d Means within a row without a common superscript letter differ (p<0.05).

**Table 7:** Effects of protected fat, soybean meal, and heat-treated soybean meal on physiological parameters of Hanwoo steers in early-fattening under heat stress conditions

Period^2)^	Treatments^1)^				SEM	p-values		
	
	Control	PF	PF+SB	PF+SB+HSB		Diet	Period	Diet×period
Rectal temperature (°C)								
Mean (diet)	38.97	38.96	38.99	38.98	0.015	0.526	<0.001	0.998
0	39.30	39.31	39.36	39.34				
1st	39.28	39.27	39.33	39.30				
2nd	38.99	38.97	39.01	39.00				
3rd	38.78	38.74	38.76	38.76				
4th	38.51	38.50	38.49	38.51				
Cortisol (ng/mL)								
Mean (diet)	8.18	8.30	8.28	8.28	0.894	0.765	<0.001	0.998
0	9.97	9.85	10.06	9.93				
1st	9.34	9.60	9.49	9.72				
2nd	8.98	9.00	8.84	8.92				
3rd	6.41	6.66	6.58	6.59				
4th	6.18	6.37	6.44	6.24				
Glucose (mg/dL)								
Mean (diet)	79.71	79.46	80.22	79.16	0.629	0.687	<0.001	0.999
0	72.32	71.54	72.23	70.32				
1st	75.71	75.59	76.27	75.30				
2nd	82.01	81.14	82.62	81.69				
3rd	83.03	83.73	83.51	83.69				
4th	85.46	85.27	86.48	84.82				
Non-esterified fatty acid (μEq/L)								
Mean (diet)	215.5	209.6	215.4	215.5	2.928	0.438	<0.001	0.992
0	261.0	257.6	264.7	260.8				
1st	237.5	242.4	236.7	241.3				
2nd	243.6	231.1	236.2	241.9				
3rd	170.7	163.3	177.0	180.2				
4th	164.8	153.6	162.5	153.3				
Blood urea nitrogen (mg/dL)								
Mean (diet)	17.39	17.45	17.43	17.31	0.118	0.829	<0.001	0.999
0	18.61	18.72	18.49	18.27				
1st	18.46	18.49	18.74	18.36				
2nd	16.89	16.94	16.92	16.91				
3rd	16.89	16.94	16.92	16.91				
4th	16.09	16.14	16.09	16.09				
Glutamic oxaloacetic transaminase (U/L)								
Mean (diet)	80.83	80.80	80.78	80.44	0.486	0.931	<0.001	0.967
0	85.73	86.40	85.98	86.36				
1st	86.46	85.48	87.40	84.97				
2nd	78.52	78.46	76.62	78.24				
3rd	76.10	78.02	76.89	75.82				
4th	77.33	75.64	77.02	76.80				

PF, protected fat; SB, soybean meal; HSB, heat-treated soybean meal; SEM, standard error of the means; RDP, rumen degradable protein; RUP, rumen undegradable protein; TDN, total digestible nutrient; CP, crude protein.

1) Control, no supplements, RDP:RUP = 62:38; PF, 10% more TDN with PF, RDP:RUP = 62:38; PF+SB, 10% more TDN and CP with protected fat and SB, RDP:RUP = 62:38; PF+SB+HSB, 10% more TDN and CP with PF, SB, and HSB, RDP:RUP = 48:52.

2) 0 period, measured on Jul 7; 1st period, Aug 11; 2nd period, Sep 8; 3rd period, Oct 6; 4th period, Nov 3.

**Table 8:** Observation of animal behaviors (min/d) in early-fattening period of Hanwoo steers fed supplemental protected fat, soybean meal, and heat-treated soybean meal under heat stress conditions

Items^2)^	Period^1)^		SEM	p-value

	1st	3rd		
Lying				
Control	476.4^b^	652.0^a^	22.19	0.005
PF	481.1^b^	655.5^a^	20.94	0.004
PF+SB	494.1^b^	647.1^a^	22.51	0.039
PF+SB+HSB	515.0^b^	642.8^a^	18.86	0.019
Total standing^3)^				
Control	707.7^a^	335.4^b^	11.53	0.001
PF	705.8^a^	329.5^b^	15.65	0.001
PF+SB	673.3^a^	367.2^b^	24.37	0.012
PF+SB+HSB	695.5^a^	372.6^b^	28.04	0.008
Walking				
Control	18.42^b^	36.67^a^	1.966	0.003
PF	18.13^b^	30.87^a^	1.436	0.014
PF+SB	14.09^b^	33.92^a^	1.541	0.011
PF+SB+HSB	19.53^b^	33.69^a^	0.702	0.001
Eating				
Control	154.3^b^	264.0^a^	14.36	0.004
PF	155.4^b^	246.9^a^	13.77	0.021
PF+SB	155.9^b^	245.0^a^	9.558	0.005
PF+SB+HSB	146.1^b^	256.1^a^	7.638	0.001
Drinking				
Control	28.29^a^	16.52^b^	1.672	0.031
PF	30.80^a^	16.47^b^	2.100	0.009
PF+SB	31.96^a^	18.56^b^	2.245	0.027
PF+SB+HSB	31.12^a^	17.87^b^	0.890	0.001
Rumination/standing				
Control	171.7^a^	99.52^b^	8.877	0.015
PF	175.1^a^	110.5^b^	4.602	0.001
PF+SB	147.8^a^	97.97^b^	9.029	0.018
PF+SB+HSB	167.3^a^	104.2^b^	10.28	0.019
Rumination/lying				
Control	337.0^b^	524.1^a^	13.82	0.002
PF	332.2^b^	522.8^a^	17.83	0.002
PF+SB	375.6^b^	535.2^a^	21.13	0.004
PF+SB+HSB	363.9^b^	499.5^a^	15.51	0.013

SEM, standard error of the means; RDP, rumen degradable protein; RUP, rumen undegradable protein; TDN, total digestible nutrient; CP, crude protein; THI, temperature-humidity index.

1) 1st period, moderate; 3rd period, comfort; observed when the same THI level lasted more than 5 days, respectively.

2) Control, no supplements, RDP:RUP = 62:38; PF, 10% more TDN with PF, RDP:RUP = 62:38; PF+SB 10% more TDN and CP with protected fat and SB, RDP:RUP = 62:38; PF+SB+HSB, 10% more TDN and CP with protected fat, SB, and HSB, RDP:RUP = 48:52.

3) Include eating, rumination, walking and drinking.

a,b Means within a row in a same parameter that do not share a letter differ (p<0.05).
